# Volume-based non-continuum modeling of bone functional adaptation

**DOI:** 10.1186/1742-4682-2-6

**Published:** 2005-02-28

**Authors:** Zhengyuan Wang, Adrian Mondry

**Affiliations:** 1 Medical and Clinical Informatics Group, Bioinformatics Institute, #07-01 Matrix, 30 Biopolis Street, 138671 Singapore

## Abstract

**Background:**

Bone adapts to mechanical strain by rearranging the trabecular geometry and bone density. The common finite element methods used to simulate this adaptation have inconsistencies regarding material properties at each node and are computationally demanding. Here, a volume-based, non-continuum formulation is proposed as an alternative. Adaptive processes corresponding to various external mechanical loading conditions are simulated for the femur.

**Results:**

Bone adaptations were modeled for one-legged stance, abduction and adduction. One-legged stance generally results in higher bone densities than the other two loading cases. The femoral head and neck are the regions where densities change most drastically under different loading conditions while the distal area always contains the lowest densities regardless of the loading conditions. In the proposed formulation, the inconsistency of material densities or strain energy densities, which is a common problem to finite element based approaches, is eliminated. The computational task is alleviated through introduction of the quasi-binary connectivity matrix and linearization operations in the Jacobian matrix and is therefore computationally less demanding.

**Conclusion:**

The results demonstrated the viability of the proposed formulation to study bone functional adaptation under mechanical loading.

## Background

Much research effort has been devoted to understanding the functional adaptation of bone under physiological loading ever since the idea of bone functional adaptation was proposed by Wolff more than one hundred years ago [1-14]. Various computational models have been put forward in the past decades and the methods describing the changing rate of bone density corresponding to strain energy density, with finite element implementation, have become the most popular of them [6,15-29].

The common finite element approach is to take the element densities as the state variables and define elements with either constant or varying densities, then update the material densities for the next step of computation according to the computed strain energy density [22,23,26,27]. With more and more powerful desktop computers and commercial finite element analysis software available, this approach is widely used today. Yet some specific problems of this approach are not well addressed so far, although decades have passed, and the numerical results are inevitably affected.

One common problem is the inconsistency of material densities on element boundaries [30]. During the updating of material densities in each step, different elements may take different densities due to strain energy density, thus often leading to conflicting material properties at the boundaries shared by more then one element. Since this conflict affects the integration points, which always come from the element boundaries, the errors are carried forward and cannot be eliminated by smoothing techniques. So it is not surprising that, if the program is allowed to run for a certain time, most of the elements tend to become either saturated or completely resorbed, leading to checker-board patterns especially in the proximal area of the femur [30].

Some effort has been made trying to solve this problem. For example, a node-based variant of the finite element method was tried with focus on the densities of the nodes rather than densities of the whole elements [21,30]. The node densities are then interpolated across the whole elements before the next step of computing begins. The results are improved, but the stress and strain quantities are still conflicting at the nodes.

Other previous work has used Voronoi structures [31] to study the effects of crack growth on trabecular bone, tapered strut models [32] to study the ageing effect through a parametric approach or continuum FEM [33] to compute the strain energy density in order to overcome individual drawbacks of the common method described. Their potential impact on the formula proposed here is discussed below.

The long existing problems and the limitations of assuming a continuum drive this new effort to explore the possibility of a non-continuum formulation of bone functional adaptations through nodal analysis in the hope of eliminating the errors present in the previous approaches. In the proposed non-continuum formulation, neighboring nodes are connected by struts that are defined with invariant material densities with respect to time but strut volumes are defined as state variables indicating different configurations of bone structure. The updating of strut volume will depend on the strain energy density in the strut in the previous step. As a result, there is no conflict either in density or in strain energy density. The shift of state variables from bone densities to bone volumes not only eliminates the errors inherent to the density-based finite element approaches but also transforms the continuum formulation to a non-continuum formulation [34]. The advantages of a volume-based non-continuum formulation may be appreciated by looking at the bone volume ratios in osteoporotic bones. The ever-increasing resolution of modern imaging techniques now allows to take a much closer look at the trabecular structure of the bone. In the trabecular network, trabeculae with different lengths and thicknesses are connected with each other to form a scaffold serving both mechanical and biological functions [35,36]. They are well connected in normal bones but poorly connected in osteoporotic bones in addition to reduced thickness. To characterize the trabecular structure, two terms are often used: bone volume/tissue volume (BV/TV) ratio and bone material orientation [15,25]. Although the cortical bone is densely packed with mineralized material, the trabecular bone dominates the inside space of the bone, highly exposed to bone marrow, highly distributed in volume, and highly involved in bone remodeling. The ratio of trabecular bone volume over tissue volume can be below 30% in osteoporotic bones, which means most space is taken up by void or bone marrow and this questions the appropriateness of a continuum formulation [35]. Besides elimination of the errors mentioned earlier, the small physiological range of bone deformation during normal activities allows linearization operations in the volume-based non-continuum formulation. This saves computation time and alleviates the high demand on hardware resources.

The proposed volume-based non-continuum formulation shows computational advantages in modeling bone functional adaptations and has much potential for clinical applications in this field.

## Results

### Changes in trabecular structure

**Figure 1:**
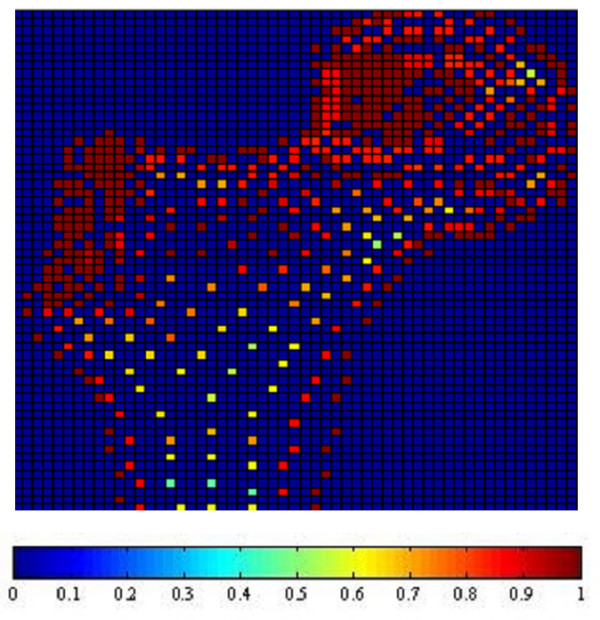
**Adaptation Results: one-legged stance. **Results of bone functional adaptation. The color bar shows percentage of actual bone density against maximum bone density, which is 1.74 g/cm^3^. The density of bone structure is not indicated by the number of sample nodes selected in that region, but by the density (converted from volume) of each node, which is expressed as degree of "red" in this illustration.

**Figure 2:**
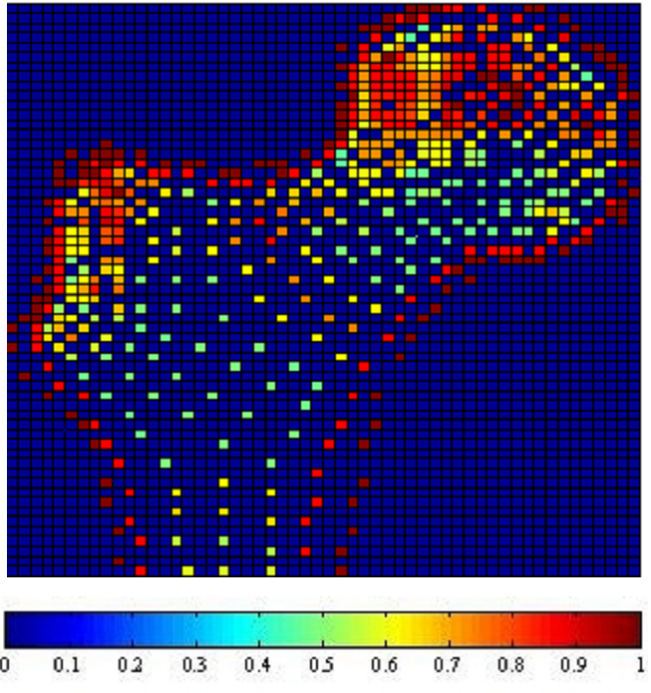
**Adaptation Results: abduction. **Results of bone functional adaptation. The color bar shows percentage of actual bone density against maximum bone density, which is 1.74 g/cm^3^. The density of bone structure is not indicated by the number of sample nodes selected in that region, but by the density (converted from volume) of each node, which is expressed as degree of "red" in this illustration.

**Figure 3:**
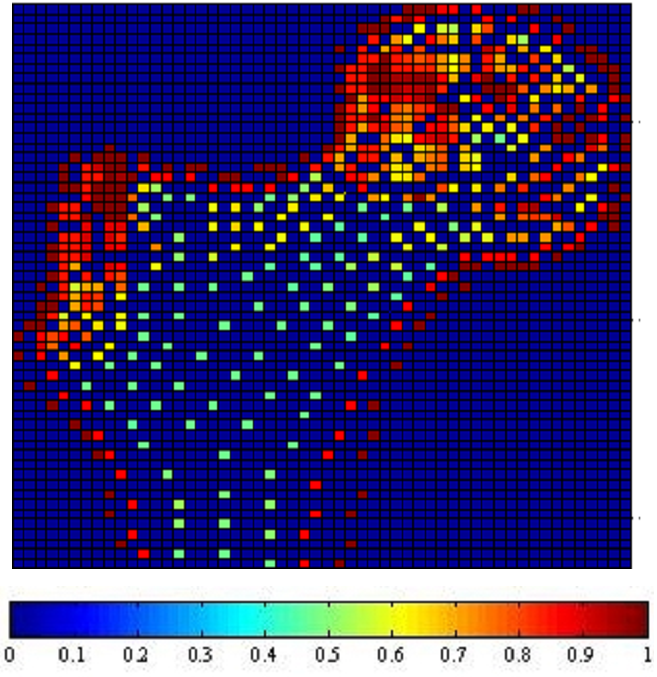
**Adaptation Results: adduction. **Results of bone functional adaptation. The color bar shows percentage of actual bone density against maximum bone density, which is 1.74 g/cm^3^. The density of bone structure is not indicated by the number of sample nodes selected in that region, but by the density (converted from volume) of each node, which is expressed as degree of "red" in this illustration.

**Figure 4:**
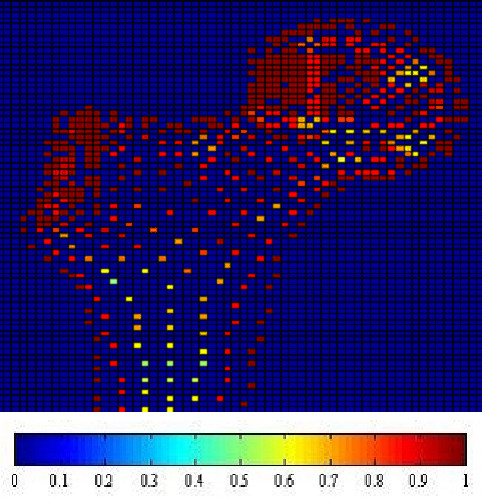
**Adaptation Results: the combined loading case. **Results of bone functional adaptation. The color bar shows percentage of actual bone density against maximum bone density, which is 1.74 g/cm^3^. The density of bone structure is not indicated by the number of sample nodes selected in that region, but by the density (converted from volume) of each node, which is expressed as degree of "red" in this illustration.

Fig. 1, 2, 3 and 4 show the node-based representation of bone adaptation results according to loading cases of one-legged stance, abduction, adduction and the combined loading case respectively.

The nodal density is a percentage relative to its maximum value (0~100%). It is 'converted' based on volume information (BV/TV) for the purpose of easy visual inspection. As suggested by Zhu [34], the value of 1.74 g/cm^3 ^is used as the maximum value in the present formulation.

In the combined loading case and one-legged stance, the nodal densities are generally high in the proximal area where a considerable number of nodes reach the highest density due to the relatively high load, while in the large distal area, the densities become lower. In the case of abduction, the external load is the smallest out of the three loading cases and the nodal density in this case seldom reaches the maximum. Although the high densities also appear in the proximal area of femoral head, the densities are lower than those of other loading cases. In the large distal area, the node densities generally range at very low levels. In the case of adduction, the highest densities still appear in the proximal area of femoral head, but in the large diaphyseal area, the densities range at very low levels.

In summary, one-legged stance and the combined loading case generally result in higher bone densities than the other two loading cases due to higher mechanical loading. The femoral head and neck are the regions where densities change most drastically under different loading conditions while the distal area always contains the lowest densities regardless of the loading conditions.

### Program convergence and bone fracture probability

**Figure 5:**
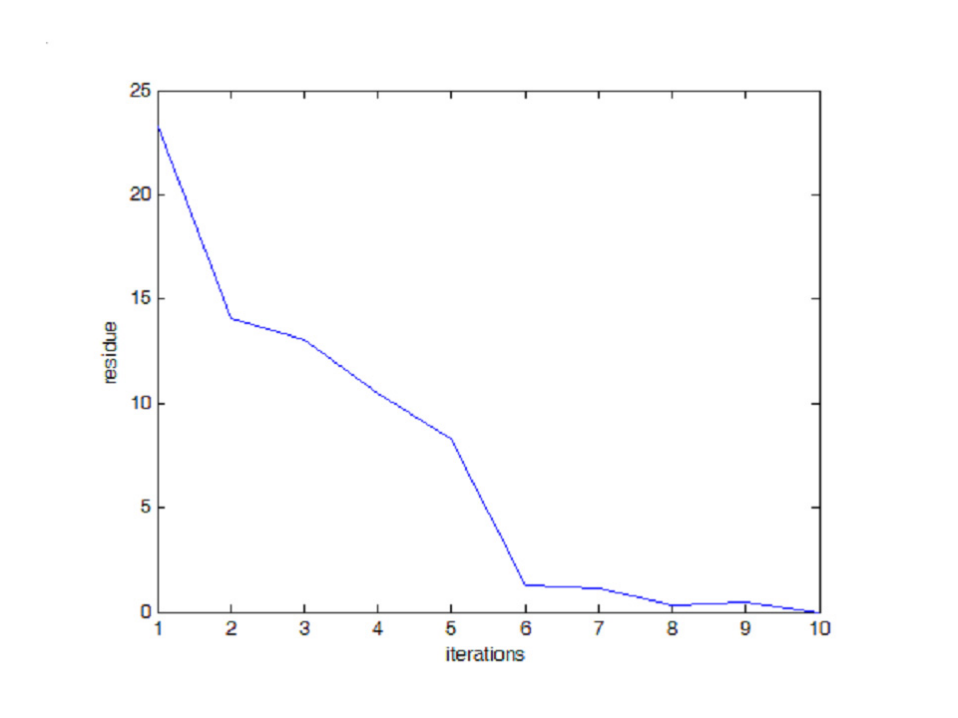
**Program Convergence. **Program convergence evaluated by residues between successive solutions.

Starting from a uniform material distribution, the program computes the strain energy density, adjusts the strut configurations, and continues on to the next iteration. Fig. 5 shows the convergence of the model, which demonstrates the adapting process of the model evaluated with the normalized error residue against the final solution. The solution vector starts with a state of uniform material distribution, it then moves toward the final state with uniform strain energy density in the solution space. As the residue of solution drops, the residue of bone volume/tissue volume ratio also drops toward trivial while the program progresses.

**Figure 6:**
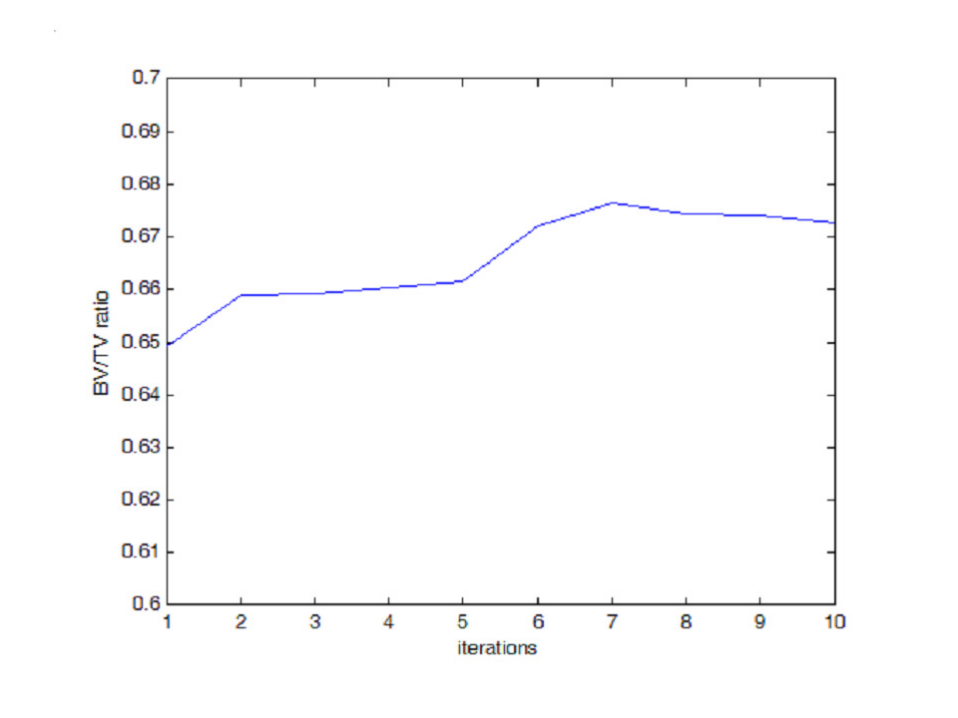
**BV/TV Evolution During Adaptation Process. **BV/TV evolution during adaptation process.

**Figure 7:**
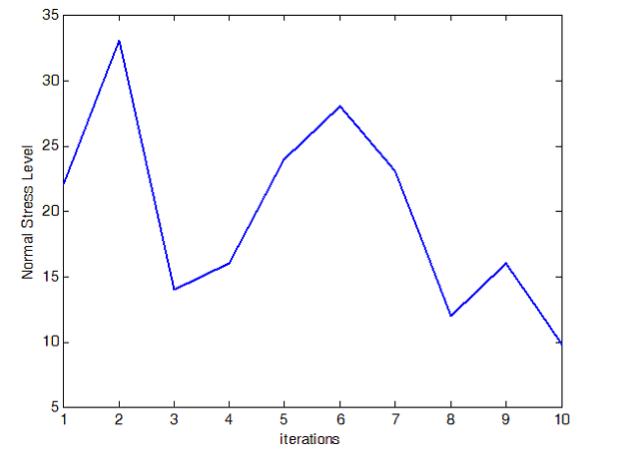
**Evolution of Apparent Normal Stress Level Within Bone Tissue. **Apparent stress level evolution during adaptation process.

**Figure 8:**
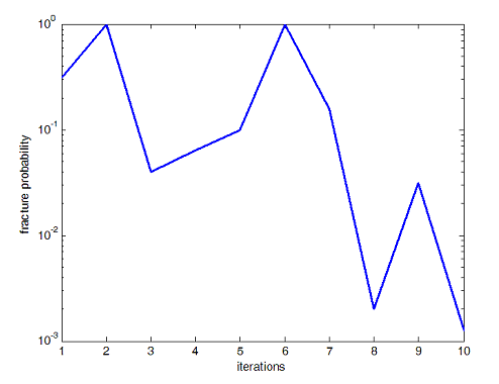
**Fracture Probability. **Bone facture probability during adaptation process.

A prediction of bone fracture risk has been proposed using stress range levels [37] from which analysis of the material fatigue strength under given loading conditions can be derived. The material is subjected to a range of stress levels due to external load, then the stress leads to fatigue damage and finally leads to collapse of the material. This prediction method was used to estimate the fracture probability of the simulation described here. The BV/TV ratios, stress levels and fracture probability are shown in Fig. 6, 7 and 8 respectively. While the program progresses toward the final solution, the volume of bone material used, indicated by the BV/TV ratio, increases by a few percents then slowly decreases again; meanwhile, the stress level moves down to a low level in the final phase and accordingly, the estimated fracture probability drops from around 90% to around 2% in the final step. It is safe to say that the program finally simulates a reasonable configuration of bone internal structure as a result of the physiological adaptation process.

## Discussion

A volume-based non-continuum formulation has been developed that describes the adaptation of bone to various mechanical loading situations. In the finite element approach to bone adaptation simulation, the integration of the entries in the coefficient matrix can be a heavy computing task [22,23,26,27]. In the model proposed here, this is alleviated through the introduction of the simpler connectivity matrix and Jacobian matrix [38]. In daily physiological activities, bone deformations are small and the Jacobian matrix can be linearized.

As mentioned in the introduction, one common problem is the inconsistency of material densities or strain energy densities on element boundaries [30]. Since all the integration points come from the boundaries, the resulting errors will essentially affect the computation. In the improved node-based implementation of the finite element method, the stress and strain quantities are still conflicting at the nodes. In the volume-based non-continuum formulation proposed here, this conflict is eliminated. The material density and strain energy density are all consistent in each individual strut, and the computing becomes less demanding.

As described above, previous work [31-33] addresses some of the weaknesses of the common FEM model. Makiyama [31] employed the "Voronoi structure" to study the effects of crack growth on trabecular bone. The method for generating a Voronoi structure could be quite useful when it calibrates the artificially constructed structure against the physical trabecular structure scanned from a patient. This might then serve as the starting state of bone configuration before adaptation begins. Moore [39] proposed a model to replace the partially damaged trabecula with another trabecula reduced in thickness. If this concept is combined with that of the strut structure, one may also derive the model proposed here, that is, a strut model with either varied modulus due to bone mineralization or adaptive cross-section/volume or even tapered struts as proposed by Kim [32].

Hip fracture is one typical manifestation of osteoporosis, and the results obtained by the simulation indicate that considerable changes of bone structure take place in the regions of femoral head and neck, where the stress level is normally higher than that of distal regions. The variations in stress level as shown in Fig. 7 reflect the adaptive process of the bone internal structure and different structural configurations will yield different stress levels in spite of little change in bone volume / tissue volume ratio.

In the current literature, the time scale for adaptive processes is not very well defined. This general lack of knowledge poses a problem for any experimental proof of concept – while the numbers of strain repetition can be predefined, they must be done within biologically suitable time frames. If a given strain comes too sudden, the bone may break instead of remodel; if the strain is applied over too long a period, it may not be a sufficient stimulus to activate adaptive processes. The lack of well-defined temporal constraints, however, is common. Kim's approach [32] is very interesting in as much as it may allow to integrate the effect of time in the model proposed here. At present, however, there is not sufficient data available to allow to integrate time effects of the clinically interesting mid-range scale, i.e. weeks to months. Kim's model looks at the process of ageing of 35 years and more.

The simulation model presented here may, beyond theoretical calculations, be applied to look at two clinical questions. Firstly, the simulation can be adjusted so that a realistic density distribution is the starting point, and outcomes following certain loading conditions, such as a predefined number of load cycles can then be predicted. Secondly, the program can integrate the measured bone density of a given patient to estimate the fracture risk based on stress level calculations.

## Conclusion

By eliminating the common inconsistencies at each node, the formulation presented here shows good numerical performance and successfully predicts reasonable bone structure changes under different loading conditions. It is viable to serve as an alternative method apart from the traditional finite element based approached to study bone adaptations. In conclusion, the volume based non-continuum formulation is a new approach to bone adaptation study and has its own advantages.

## Methods

### Volume-based representation of the trabecular bone structure

**Figure 9:**
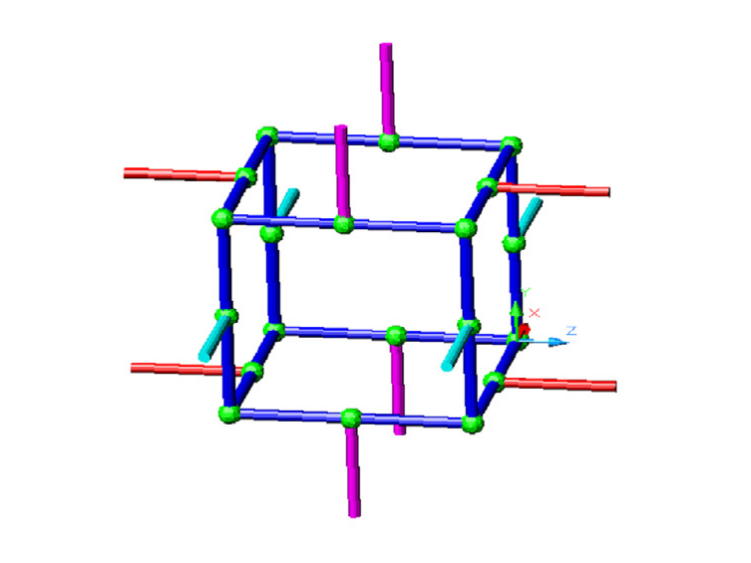
**Bone Structure Decomposition. **Representation of bone material by struts. Each strut can assume different geometric dimensions and material properties. Apparent mechanical property of bone material is based on the strut configuration.





In the volume-based non-continuum formulation used here, the trabecular structure is represented by a connected strut system and each strut can take different sizes according to the mechanical loading requirements, that is, strain energy density. The strut representation is shown in Fig. 9, which resembles a small volume of the trabecular structure. In this setting, the BV/TV ratio can be directly obtained from the ratio of the strut volumes over the unit volume, and material orientation  can be obtained though the resultant of the vectorial material components of the struts, as described by equation (1) and (2).

where *v*_*i *_is the volume of the *i*-th strut in the *j*-th basic unit, *V*_*j *_is the volume of the *j*-th basic unit, *R*_*j *_is the material orientation of the *j*-th basic unit,  is the orientation of the *i*-th strut in the *j*-th basic unit and *N *is the number of struts in the *j*-th basic unit.

For the formulation proposed here, the bone structure is decomposed into and represented by a connected network of struts. These struts are a mathematical abstraction of the physical bone structure, from which the BV/TV ratio can be derived. The thickness of a strut is adapted during the bone adaptation process in mimicry of the physiological processes.

### Volume adaptation under mechanical loading

The bone mass will vary under mechanical loading. In engineering, the general relationship between varying mass, density and volume is described as:



Since the density here is taken as constant regarding time, the second term on the right hand side, , simply vanishes and the mass variation is realized through volume variation under mechanical loading. Based on the density-based adaptation proposed by Zhu X. *et al *(39), which is stated as:



the volume-based adaptation can thus be stated as:



where *β*_*i *_= *U*_*i *_/ *ρ*_*i*_*k*, which is a comparative coefficient describing the comparison of a given mechanical stimulus in each sensor cell with the reference value *k*, and *U*_*i *_represents the strain energy density for the *I*-th sensor unit; *N *is the number of sensor cells and *f*_*i*_(*x*) is the spatial influence function; *B*(*t*) is a remodeling coefficient; *α *indicates the remodeling power of strain energy density [34].

### Non-Continuum formulation

With the whole bone represented by a volume-based strut system, the non-continuum formulation can be noted as follows:



where *A *is a connectivity matrix describing the connecting relationship between the struts, *α *is the linearized Jacobian matrix,  is the nodal displacement vector to be solved for and  is the loading vector derived from the external mechanical load. The generalized conjugate residue method is used to solve this formulation [38,40].

**Figure 10:**
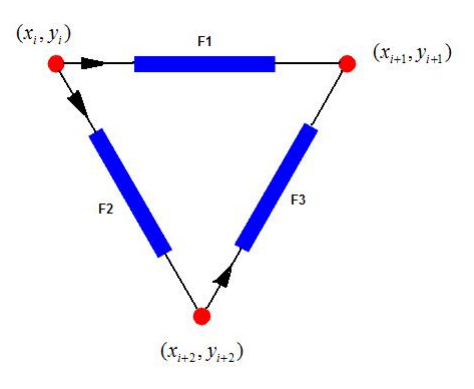
**Connectivity of Struts. **Physical connectivity relationship between struts is indicated by the connectivity matrix.



Connectivity matrix A is the matrix to show the relationship between connected struts with the entries of 1, -1 or 0. A strut starts from the node with the index corresponding to the entry 1 and ends at the node with the index corresponding to -1. It is further illustrated in Fig. 10 and equation (7).

**Figure 11:**
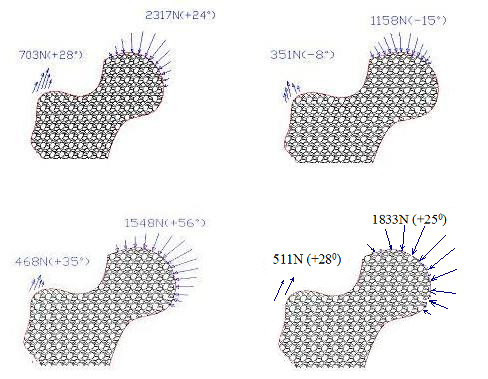
**Loading cases. **Quantitative information of different loading cases. Four loading cases are considered: one-legged stance, abduction, adduction and the combined loading case (weighted based on their respective daily occurrence cycles).

Finally, the different loading conditions to be applied are shown in Fig. 11.

## Competing interests

The author(s) declare that they have no competing interests.

## Authors' contributions

Zhengyuan Wang developed the formulation and partly prepared the manuscript, Adrian Mondry participated in the adaptation controls and partly prepared the manuscript.
